# Design of CoMoCe-Oxide Nanostructured Composites as Robust Bifunctional Electrocatalyst for Water Electrolysis Overall Efficiency

**DOI:** 10.3390/ma18174052

**Published:** 2025-08-29

**Authors:** Akbar I. Inamdar, Amol S. Salunke, Jyoti V. Patil, Sawanta S. Mali, Chang Kook Hong, Basit Ali, Supriya A. Patil, Nabeen K. Shrestha, Sejoon Lee, Sangeun Cho

**Affiliations:** 1 Division of System Semiconductor, Dongguk University, Seoul 04620, Republic of Korea; 2 Department of Semiconductor Science, Dongguk University, Seoul 04620, Republic of Korea; 3 Polymer Energy Materials Laboratory, School of Chemical Engineering, Chonnam National University, Gwangju 61186, Republic of Korea; 4 Optoelectronic Convergence Research Center, School of Chemical Engineering, Chonnam National University, Gwangju 61186, Republic of Korea; 5 Department of Chemistry and Materials Science, School of Chemical Engineering, Aalto University, P.O. Box 16100, FI-00076 Espoo, Finland; 6 Department of Nanotechnology and Advanced Materials Engineering, Sejong University, Seoul 05006, Republic of Korea

**Keywords:** ternary oxide, morphology control, electrocatalyst, bifunctional electrolysis, overall water splitting

## Abstract

The development of ternary metal oxide electrocatalysts with optimized electronic structures and surface morphologies has emerged as one of the effective strategies to improve the performance of electrochemical water splitting. In this work, ternary CoMoCe (CMC)-oxide electrocatalysts were successfully synthesized on nickel foam substrates via a hydrothermal technique and employed for their catalytic activity in an alkaline electrolyte. For comparison, binary counterparts (CoMo, CoCe, and MoCe) were also fabricated under similar conditions. The synthesized catalysts’ electrodes exhibited diverse surface architectures, including microporous-flake hybrids, ultrathin flakes, nanoneedle-assembled microspheres, and randomly oriented hexagonal structures. Among them, the ternary CoMoCe-oxide electrode exhibited outstanding bifunctional electrocatalytic activity, delivering low overpotentials of 124 mV for the hydrogen evolution reaction (HER) at −10 mA cm^−2^, and 340 mV for the oxygen evolution reaction (OER) at 100 mA cm^−2^, along with excellent durability. Furthermore, in full water-splitting configuration, the CMC||CMC and RuO_2_||CMC electrolyzers required cell voltages of 1.69 V and 1.57 V, respectively, to reach a current density of 10 mA cm^−2^. Remarkably, the CMC-based electrolyzer reached an industrially relevant current density of 1000 mA cm^−2^ at a cell voltage of 2.18 V, maintaining excellent stability over 100 h of continuous operation. These findings underscore the impact of an optimized electronic structure and surface architecture on design strategies for high-performance ternary metal oxide electrocatalysts. Herein, a robust and straightforward approach is comprehensively presented for fabricating highly efficient ternary metal-oxide catalyst electrodes, offering significant potential for scalable water splitting.

## 1. Introduction

With rapid technological advancements, the global demand for clean energy has grown substantially. Currently, most of the energy demands are fulfilled by non-renewable energy sources such as oil, coal, and natural gas. However, these fossil fuels are not only limited in supply but also significantly responsible for environmental pollution. In response to the climate crisis, the global community has committed to achieving carbon neutrality (net-zero emissions) by 2050 [1,2,3], presenting an urgent challenge to transition to clean and sustainable energy sources. Accordingly, growing concerns over the current energy landscape and increasing demand for renewable energy sources have driven researchers to explore energy-efficient and environment-friendly alternatives to fossil fuels [4]. Hydrogen produced via electrochemical water splitting has recently emerged as a promising alternative energy source to fossil fuels [5,6]. It possesses several outstanding properties, including zero carbon emissions, high energy efficiency, and the potential to supply up to 75% of future global energy needs [7]. The green hydrogen produced via renewable energy-driven water electrolysis is increasingly recognized as a key technology for achieving global net-zero emissions. In this process, water electrolysis involves two half-cell reactions: the oxygen evolution reaction (OER) at the anode and the hydrogen evolution reaction (HER) at the cathode, both of which require highly efficient electrocatalyst electrodes. Over the past two decades, significant efforts have been devoted to developing efficient electrocatalyst electrodes for hydrogen production, including those based on precious metals such as Pt, Ru, and Ir [8,9,10,11]. However, the scarcity and high cost of these precious metals hinder their large-scale deployment, prompting the search for cost-effective and earth-abundant alternatives.

To address challenges, recent research has focused on binary and ternary metal oxides, which have demonstrated outstanding electrochemical properties for OER, HER, and overall water splitting (OWS) [12,13,14,15,16,17,18,19,20,21]. For instance, a catalyst electrode of heterogeneous core–shell CoFeMo polymetallic sulfide/N-rich carbon was fabricated to improve catalytic activity via optimizing its electronic structure and morphology, allowing for larger surface area, electrical conductivity, and catalytic stability. This system achieved OWS at a cell voltage of 1.76 V to reach a current density of 100 mAcm^−2^ [12]. Similarly, sputtered ternary NiFeCo metal oxide catalysts exhibited a low OER overpotential value (248 mV at a current of 10 mA cm ^−2^) and excellent durability over 500 h in an alkaline electrolyte, highlighting the significance of optimized synthesis strategies and substrate selection [13]. In another example, NiFeMo layered double hydroxide nanoflakes synthesized via a simple hydrothermal technique possessed overpotentials of 200 mV (OER) and 86 mV (HER) to reach 10 mA cm^−2^ [14]. The industrially relevant current density of 1000 mA cm^−2^ is obtained at only 2.1 V with excellent stability over 90 h at high currents. Theoretical analysis suggests that the Mo and Fe are the main active sites for HER and OER processes, respectively.

Several other binary and ternary oxides such as NiFe_2_O_4_, MoO_x_/CoMo on Cu nanowires, CoO/MoO_x_ crystalline/amorphous nanostructures, CoMo layered double hydroxides (LDHs), and bifunctional CoMo electrocatalysts also investigated for water splitting activity [15,16,18,19,20,21]. Their superior bifunctional activity is attributed to the synergistic effects from multi-metal interactions, high surface area, and high electrical conductivity of multi-metal oxides. In particular, precise compositional tuning via atomic layer deposition has enabled optimized binding energies of the catalysts, thereby improving their OER activity. For example, the electrodeposited MoO_x_/CoMo on Cu nanowires showed excellent HER (56 mV) and OER (211 mV) activity in an alkaline medium. The lowest OWS at 1.45 V was obtained with a stability of 10 h at a current density of 50 mA cm^−2^. Another strategy of heterogeneous CoO/MoO_x_ fabrication is also an effective way to display superior catalytic activity, benefiting from the large defect-rich interface. Moreover, LDHs are also excellent candidates for bifunctional OER/HER activity with outstanding electrochemical performances. Thus, the development of binary and ternary metal oxides is one of the effective ways to achieve highly efficient bifunctional catalysts.

In this work, we synthesized ternary CoMoCe-oxide catalyst electrodes on a nickel foam (NF) substrate via a hydrothermal method. The electrochemical performance of CoMoCe-oxide was systematically investigated and compared with its binary counterparts (CoMo-oxide, CoCe-oxide, and MoCe-oxides). The resulting catalysts exhibited distinct morphologies depending on their compositions. Due to the enlarged electrochemical surface area and enhanced electrical conductivity, the CoMoCe-oxide electrode demonstrated excellent catalytic activity, achieving low overpotentials of 124 mV (HER) and 340 mV (OER) at current densities of −10 and 100 mA cm^−2^, respectively. Moreover, the overall water splitting activity of the CoMeCe-oxide electrode coupled with commercial RuO_2_ was achieved at a cell voltage of only 1.57 V to reach 10 mA cm^−2^. These results highlight the potential of CoMoCe-oxide as an efficient and cost-effective catalyst for sustainable hydrogen production.

## 2. Experimental Section

### 2.1. Materials

All chemicals and reagents were purchased from Sigma-Aldrich and used without any further purification. The reagents included cobalt (II) nitrate hexahydrate [Co(NO_3_)_2_·6H_2_O], ammonium molybdate tetrahydrate [(NH_4_)_6_Mo_7_O_24_·4H_2_O], cerium (III) nitrate hexahydrate [Ce(NO_3_)_3_6H_2_O], and urea [CO(NH_2_)_2_].

### 2.2. Synthesis of Trimetallic CoMoCe-Oxide Catalysts

Trimetallic CoMoCe-oxide catalyst electrodes were synthesized on NF substrates via a simple hydrothermal method. Prior to deposition, the NF substrates were cleaned with dilute acid to remove surface impurities. To fabricate trimetallic CoMoCe-oxide thin-film electrodes, a cobalt nitrate, ammonium molybdate tetrahydrate, and cerium nitrate were dissolved in deionized (DI) water in a 1:1:1 molar ratio. Urea was then added to the solution at a concentration of 0.3 M. The resulting precursor solution was transferred to a Teflon-lined stainless-steel autoclave, and a precleaned NF substrate was immersed in it. The autoclave was sealed and heated at 150 °C for 12 h in the muffle furnace. After cooling to room temperature naturally, the resulting CoMoCe-oxide-coated NF substrate was then thoroughly washed with DI water, methanol, and air-dried at ambient conditions. For comparison, binary compositions including CoMo-, CoCe-, and MoCe-oxides were also synthesized using the same hydrothermal procedure, with 1:1 molar ratios of the respective metal precursors. For simplicity, the synthesized CoMoCe-, CoMo-, CoCe-, and MoCe-based oxides are hereafter denoted as CMC, CM, CC, and MC, respectively.

### 2.3. Characterization and Electrochemical Measurements

The phase composition of the synthesized catalysts was analyzed by X-ray diffraction (XRD) using a Bruker D2 phaser instrument with Cu-Kα radiation (λ = 1.5406 Å). Phase identification was performed using standard PDF-5+ ICDD database (licensed by Aalto University). Raman analysis was carried out using RENISHAW Raman microscope (New Mills, UK). Surface morphology and elemental composition were examined by field-emission scanning electron microscopy (FE-SEM, JSM-IT200, JEOL, Tokyo, Japan), equipped with energy-dispersive spectroscopy (EDS, JEM-2100F, JEOL, Japan). X-ray photoelectron spectroscopy (XPS, K-α Thermo Scientific, Waltham, MA, USA) was employed to analyze the chemical bonding states of the constituent elements. The electrochemical measurements were conducted using a Bio-Logic MPG-2 (Seyssinet-Pariset, France) electrochemical workstation. A three-electrode cell consisting of a saturated calomel electrode (SCE) was used as a reference electrode, Pt (for OER) and Graphite (for HER) as counter electrodes, and fabricated catalyst electrodes were working electrodes. For all the electrochemical experiments an alkaline electrolyte of 1 M KOH was used. The stability of the CoMoCe was assessed using the chronoamperometric technique at a current density of 100-mA cm^−2^ at room temperature in 1 M KOH electrolyte. The OWS activity was evaluated in a two-electrode configuration, with the catalyst electrodes serving as both the anode and cathode.

## 3. Results and Discussion

**Figure 1:**
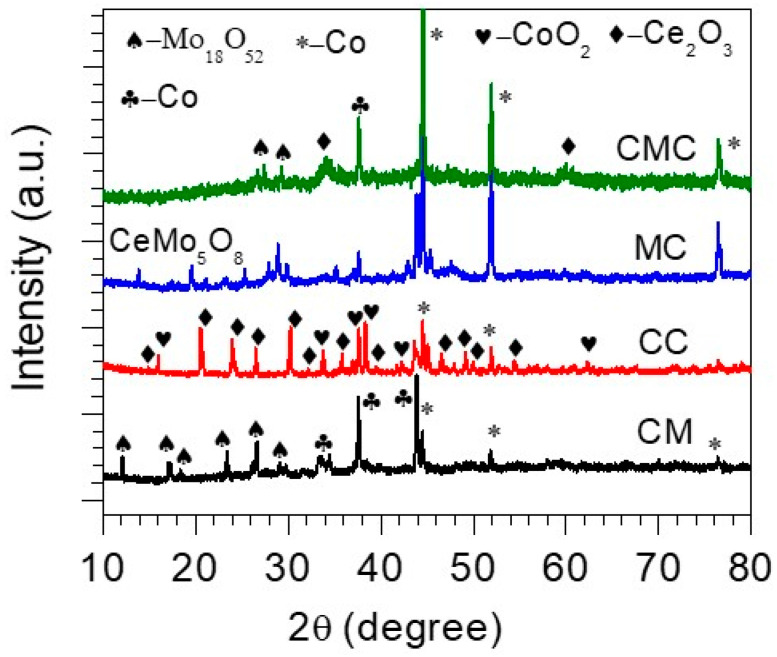
XRD patterns of as-prepared binary CM, CC, and MC, and ternary CMC.

XRD analysis was conducted to determine the crystalline phases present in the synthesized samples. As shown in Figure 1, the XRD patterns reveal the formation of mixed-metal phases, such as binary CM, CC, and MC, and ternary CMC. Characteristic diffraction peaks corresponding to each phase were identified and matched with standard JCPDS reference cards. For the CM phase, distinct reflections were observed at around 2θ = 12°, 17°, 18.3°, 23.4°, 26.6°, 29.2°, 33°, 37.6°, 43.9°, 51.8°, and 76.3°, aligning with the (hkl) = (100), (112), (004), (201), (03-3), (214), (202), (004), (111), (200), and (202), respectively. These peaks match well with Mo_18_O_52_ (JCPDS # 96-153-7520) and Co phases (JCPDS # 96-901-2950 and # 96-410-5685). The CC and MC samples exhibited peaks attributable to Ce_2_O_3_ (JCPDS # 96-101-0280), CoO_2_ (JCPDS # 96-153-1763), Metallic Co (JCPDS # 96-901-2950), and CeMo_5_O_8_ (JCPDS # 96-210-3925), with prominent reflections located at 2θ = 14.9°, 15.9°, 20.5°, 23.9°, 26.5°, 30.2°, 32.2°, 33.7°, 35.8°, 37°, 37.6°, 38.3°, 39.7°, 41.6°, 42.3°, 44.5°, 46.5°, 47.9°, 49°, 50°, 51.9°, 54.5°, 62.3°, and 76.6°. These values confirm the presence of the binary metal oxide phases. In the case of the ternary CMC sample, additional diffraction peaks were observed, which could be ascribed to overlapping features from the constituent binary phases, suggesting the successful formation of a multicomponent oxide phase—potentially a solid solution or alloy-type structure. Notably, no significant impurity peaks or undesired oxides were detected, indicating high phase purity. The overall diffraction profile is consistent with a polycrystalline material with well-defined peaks, suggesting good crystallinity. The main phases identified include Mo_18_O_52_ (JCPDS # 96-153-7520), Co (JCPDS # 96-901-2950), Ce (JCPDS # 96-153-3275), and Co (JCPDS # 96-410-5685), with cobalt notably existing in two different crystalline forms. Diffraction peaks marked with asterisks originate from the underlying NF substrate. The XRD analysis shown in Figure 1 reveals that cobalt appears in two different crystalline forms: metallic fcc-Co (JCPDS #96-901-2950), which has reflections at about 2θ ≈ 43.9° and 51.8°, and metallic hcp-Co (JCPDS #96-410-5685), with a reflection at 76.3°. Additionally, there are phases that contain cobalt oxide.

**Figure 2:**
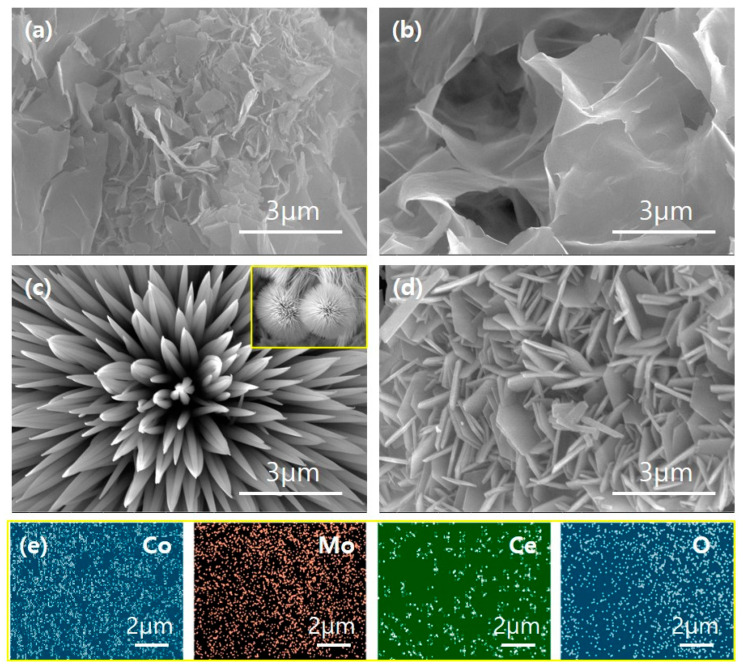
Scanning electron microscopic images of the as-prepared (**a**) CMC, (**b**) CM, (**c**) CC, inset shows the enlarged view of the microspheres and (**d**) MC, plus (**e**) EDS elemental mapping images of ternary CMC catalyst electrodes, revealing the presence of the Co, Mo, Ce, and O in the sample.

The surface morphologies of the CMC and other binary samples were investigated using FE-SEM, as shown in Figure 2a–d. All the samples exhibited different surface morphologies and shapes depending on their constituent elements. The ternary CMC sample displayed a mixed structure of micropores and flakes (Figure 2a and Figure S1a). In contrast, the CM sample formed ultrathin flakes (Figure 2b and Figure S1b), the CC sample showed microspheres embedded with nanoneedles (Figure 2c and Figure S1c), and the MC sample exhibited randomly oriented hexagonal plates (Figure 2d and Figure S1d). These morphological differences suggest significant variations in electrochemically active surface areas, which could influence the catalytic performance. To confirm the elemental composition and distribution of the CMC, energy-dispersive X-ray spectroscopy (EDS) mapping was performed (Figure 2e and Figure S2a,b). The mapping images clearly confirm the uniform and homogeneous distribution of Co, Mo, Ce, and O across the catalyst surface, validating the successful incorporation of all target elements.

**Figure 3:**
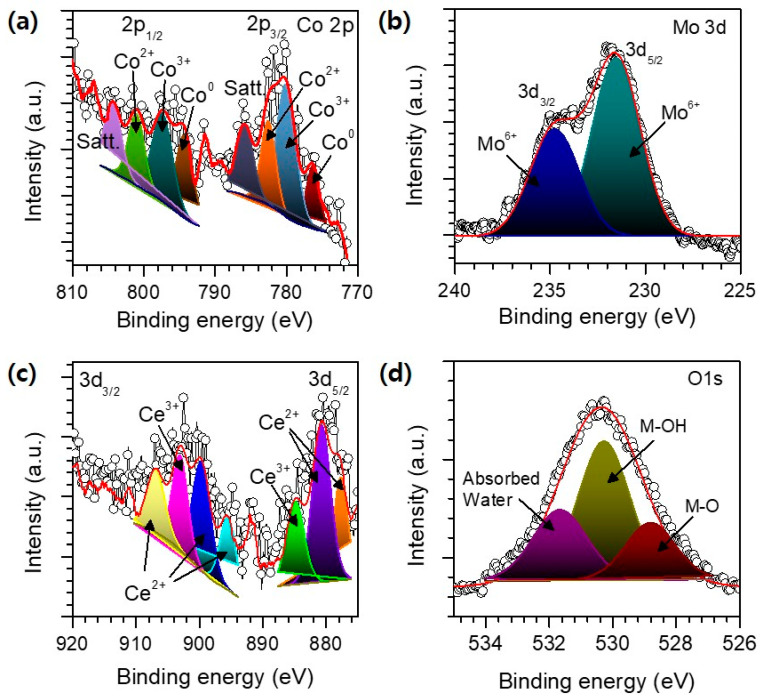
XPS analysis of optimized CMC sample. Black line is experimental data and red line is fitted data. High resolution XPS spectra of (**a**) Co 2p, Dark gray and purple peaks are satellite peaks (**b**) Mo 3d, (**c**) Ce 3d, and (**d**) O1s.

The chemical oxidation states of the CMC were investigated via XPS analysis. The XPS survey spectra of (Figure S3) confirmed the presence of Co, Mo, Ce, O and C in the sample. The core level 2p_3/2_ and 2p_1/2_ spectra of the Co 2p (Figure 3a) exhibited major peaks at binding energies of 780.14 eV and 794.2 eV, corresponding to Co^3+^, and at 782.54 eV and 797.5 eV were associated with Co^2+^ [22,23,24,25]. Two additional peaks at 785.89 eV and 801.1 eV were assigned to the satellite peaks of 2p_3/2_ and 2p_1/2_, respectively. The metallic Co was also recognized at 776 and 794 eV. The high-resolution Mo 3d XPS spectra (Figure 3b) exhibited two peaks at binding energies of 231.7eV and 234.8 eV which are associated with 3d_5/2_ and 3d_3/2_, respectively, suggesting the existence of Mo^6+^ oxidation states [26,27]. The core level XPS spectrum of Ce 3d (Figure 3c) revealed seven peaks, which are associated with the Ce 3d_5/2_ and Ce 3d_3/2_ spin-orbit splitting, depicting the presence of the Ce^3+^ and Ce^4+^ oxidation states. The peaks at 884.9 eV and 903.1 eV are assigned to Ce^3+^ while other peaks correspond to Ce^4+^, confirming the coexistence of mixed oxidation states [28,29,30]. The O 1s spectrum show the peaks at the binding energies of the 528.7 eV, 530.2 eV, and 531.69 eV are associated with metal oxide, lattice oxygen, and loosely bound adsorbed oxygen, respectively [31]. These results confirm the successful incorporation of the intended elements with their expected oxidation states, which may contribute to the enhanced electrochemical performance of the catalyst. The contradiction in the oxidation state observed from the XRD and XPS results could be due to the differing sampling depths of the two methods. XRD looks at bulk crystalline phases, whereas XPS is sensitive to the surface, analyzing just the top ~5 nm and thus primarily detecting oxidized surface species. These findings imply that while metallic cobalt can exist in various bulk crystal structures, it tends to be partially oxidized at the surface during the synthesis process or when exposed to air.

**Figure 4:**
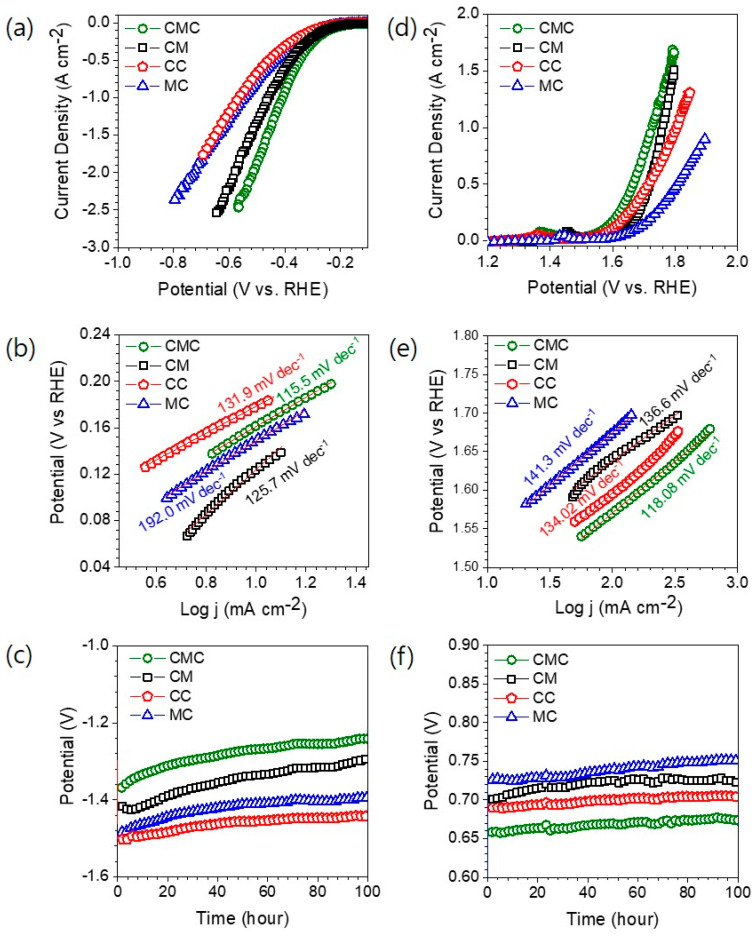
(**a**) iR-corrected HER polarization curves recorded at scan rate of 5 mVs^−1^ in 1 M KOH electrolyte. (**b**) HER Tafel slopes. (**c**) Chronoamperometric stability curves for HER at current density of −100 mA cm^−2^ in 1M KOH electrolyte at room temperature. (**d**) iR-corrected OER polarization curves. (**e**) OER Tafel slopes. (**f**) Chronoamperometric stability curves for OER at 100 mA cm^−2^ in 1M KOH electrolyte at room temperature.

Electrochemical measurements for both HER and OER were carried out using a standard three-electrode setup in 1 M KOH at room temperature. Prior to linear sweep voltammetry (LSV) measurements, all electrodes were activated via 50 cycles of cyclic voltammetry (CV) at a scan rate of 100 mV s^−1^ to ensure surface stabilization and reproducibility. Later, iR-corrected LSV measurements were conducted at a scan rate of 5 mV s^−1^ to evaluate the HER performance (Figure 4a). The LSV curves clearly demonstrate composition-dependent variations in electrocatalytic performance, highlighting the synergistic effects among the incorporated metal specie. The integration of Co, Mo, and Ce significantly influenced the catalytic activities in their oxide compounds, as reflected in the HER activity across the applied voltage range. Each catalyst exhibited distinct HER activity, indicating that the variation in metal content plays a critical role in tuning the electrochemical properties. Among all samples, the ternary CMC displayed the most superior HER performance, requiring only a small overpotential of 124 mV to achieve a current density of −10 mA cm^−2^, significantly lower than that of CM (149 mV), CC (178 mV), and MC (162 mV). In addition, CMC maintained a high current density of 1000 mA cm^−2^ at a minimal overpotential of 407 mV. The HER overpotential obtained for the CMC catalyst was notably lower than those of several previously reported HER electrocatalysts, underscoring its exceptional catalytic activity.

The CMC system demonstrated superior HER performance compared to several state-of-the-art electrocatalysts reported in the literature, including H–Fe–CoMoS (137 mV) [32], CoS_2_/WS_2_ (130 mV) [33], CoMoS_4_ (143 mV) [34], MnO_2_ NSs/Co_3_O_4_ nanoparticles (129 mV) [35], Au-doped Co-Ni hydroxide (200 mV) [36], NiCo-LDH (162 mV) [37], and Co(OH)_2_@NCNTs (170 mV) [38]. Furthermore, the HER overpotentials required to achieve various current densities (10, 100, and 500 mA cm^−2^) for all synthesized catalysts are summarized in Table S1 (Supporting Information). These comparative data highlight the superior electrocatalytic efficiency and potential of the CMC catalyst for practical hydrogen evolution applications. The enhanced HER performance can be primarily attributed to the synergistic interaction among Co, Mo, and Ce, which not only increases the active surface area but also generates a greater number of accessible active sites [12,20,39]. Moreover, the formation of a heterostructured interface among the metal species in CMC likely facilitates spontaneous electron transfer, as observed in similar systems. This internal redistribution may result in the formation of electrophilic and nucleophilic surface domains, enhancing the adsorption of key reaction intermediates such as OH^−^ and H^+^ [12].

To gain deeper insight into the reaction mechanism and kinetics, additional electrochemical techniques were conducted, including Tafel slope analysis, electrochemical surface area (ECSA) estimation, and electrochemical impedance spectroscopy (EIS). The ECSA was then calculated using the following equation [40]:

ECSA = C_DL_/Cs (1)

where Cs is the specific capacitance of the electrolyte (Cs = 0.04 mF cm^−2^ for 1 M KOH). Among these, the Tafel slope is a critical kinetic parameter, reflecting both the rate-determining step and overall reaction kinetics. Derived from the LSV polarization curves using the Tafel equation, the CMC catalyst exhibits a Tafel slope of 115.5 mV dec^−1^ (Figure 4b), which is significantly lower than those of CM (125.7 mV dec^−1^), CC (131.9 mV dec^−1^), and MC (192 mV dec^−1^). This result is consistent with the LSV data and confirms that CMC possesses faster reaction kinetics compared to its binary counterparts. The relatively low Tafel slope also suggests enhanced intrinsic conductivity and optimized charge transfer pathways, reinforcing the potential of CMC as an efficient electrocatalyst for HER under alkaline environments.

As shown in Figure 4c, chronoamperometric stability tests were performed at a constant current density of 100 mA cm^−2^ for 100 h (without iR correction) to evaluate the long-term durability of the catalysts. All samples exhibited stable electrochemical performance throughout the testing period without significant degradation in activity. Among the tested samples, the CMC electrode maintained the lowest overpotential during the entire 100 h test, consistent with its superior activity observed in the LSV results. Such stable behavior at high current density confirms the excellent operational durability of the CMC catalyst. The robust performance indicates that the synergistic Co–Mo–Ce configuration promotes sustained hydrogen evolution at a low energy cost. To further validate the structural and electrochemical integrity of the CMC catalyst, LSV curves were recorded before and after the 100 h chronoamperometric test (Figure S4). The near-identical overlap of the LSV curves before and after testing confirms the excellent durability and stability of the catalyst, indicating negligible degradation in both structure and activity under prolonged HER operation. These results strongly support the suitability of CMC for long-term hydrogen evolution in alkaline environments. Figure 4d presents the iR-corrected LSV curves recorded for OER at a scan rate of 5 mV s^−1^ in 1 M KOH electrolyte. Consistent with the HER results, the CMC electrode demonstrated the best OER activity among all samples, requiring only 340 mV of overpotential to achieve 100 mA cm^−2^. In contrast, CM, CC, and MC required 411 mV, 365 mV, and 444 mV, respectively, to reach the same current density [41]. A comprehensive comparison of the OER overpotentials is presented in Table S2. Furthermore, CMC achieved a current density of 1000 mA cm^−2^ at a notably low overpotential of 496 mV, far outperforming the other compositions and confirming its superior high-current catalytic capability. To further understand the OER kinetics, Tafel slope analysis was performed based on the LSV curves (Figure 4e). The CMC displayed the lowest Tafel slope of 118.08 mV dec^−1^, compared to 136.6 mV dec^−1^ for CM, 134.02 mV dec^−1^ for CC, and 141.3 mV dec^−1^ for MC. This lower Tafel slope indicates more efficient charge transfer and faster reaction kinetics, attributable to the synergistic interactions and optimized electronic structure of the trimetallic composition. The long-term operational stability of the catalysts during OER was further examined by chronoamperometric tests at a constant current density of 100 mA cm^−2^ for 100 h. As depicted in Figure 4f, CMC maintained a stable potential throughout the entire test duration, with no significant increase in its overpotential. This excellent stability was further corroborated by post-test LSV measurements (Figure S5), which showed negligible change compared to the pre-test curves. These results confirm that the CMC catalyst not only delivers high catalytic activity but also maintains exceptional electrochemical and structural stability during prolonged OER operation, making it a promising candidate for practical water-splitting applications.

**Figure 5:**
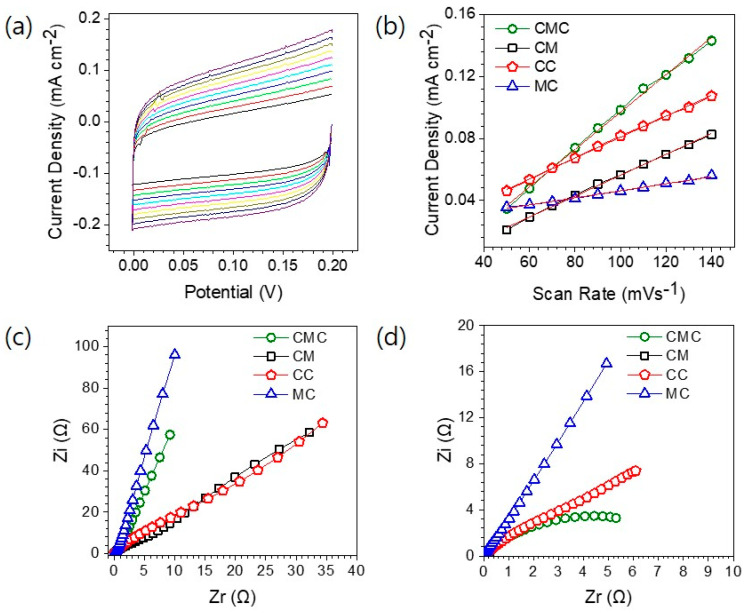
(**a**) Cyclic voltammetry curves of CMC electrocatalyst recorded in non-faradaic region at different scan rates of 50–140 mVs^−1^ for ECSA estimation. (**b**) Plot of current density (mA cm^−2^) versus scan rate at fixed non-faradaic potential (0.15 V vs. SCE). The additional red line fitting data. (**c**,**d**) Nyquist plots of all samples recorded at OCP and 0.4 V.

To gain deeper insight into the origin of the enhanced catalytic activity, ECSA and EIS measurements were conducted. The ECSA, which is directly related to the number of accessible electrochemically active sites, was estimated from the double-layer capacitance (C_DL_) values obtained through cyclic voltammetry at various scan rates between 50–140 mVs^−1^ (Figure 5a and Figure S6). As shown in Figure 5b, the current density was plotted against scan rates at a fixed non-faradaic potential (0.15 V vs. SCE), and the slope of this linear relationship yielded the C_DL_. The calculated ECSA values for all catalysts are listed in Table S3. Among the samples, CMC exhibited the highest ECSA of 30.25 cm^2^, indicating a large number of electrochemically active sites and aligning with its superior HER and OER activity. To probe the charge transfer kinetics, EIS measurements were performed both at open circuit potential (OCP) and under a working bias of 0.4 V vs SCE [9]. Nyquist plots presented in Figure 5c (at OCP) and Figure 5d (at 0.4 V) demonstrate that CMC possesses the lowest charge transfer resistance among all tested samples. The smaller semicircle observed for CMC under biased conditions indicates faster interfacial electron transfer and enhanced ion diffusion, corroborating its excellent conductivity and rapid reaction kinetics. These combined results suggest that the superior catalytic performance of the CMC system is due to the synergistic interplay of the high electrochemical surface area and low charge-transfer resistance, and the favorable electronic environment provided by the trimetallic composition.

**Figure 6:**
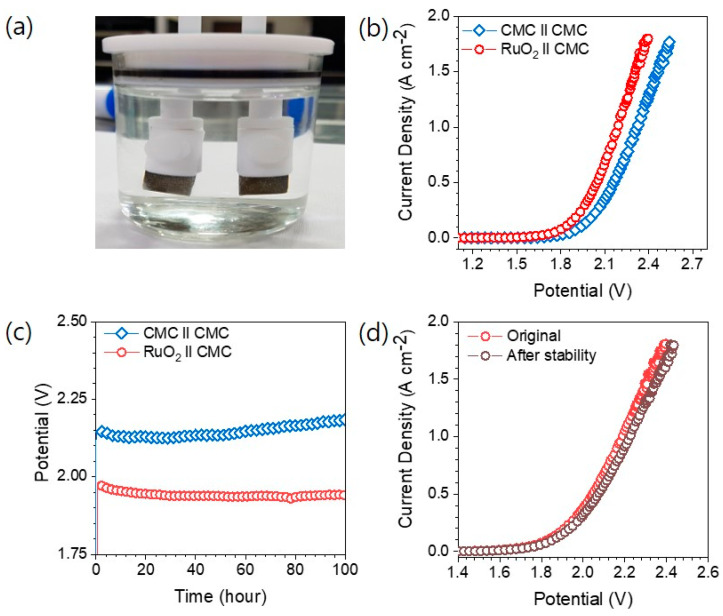
(**a**) Digital photograph of electrolyzer setup fabricated using ternary CMC electrocatalyst. (**b**) OWS polarization curves of CMC||CMC and RuO_2_||CMC systems were recorded at scan rate of 5 mVs^−1^. (**c**) Chronoamperometric stability curves for OWS at 100 mA cm^−2^ in 1M KOH electrolyte at room temperature. (**d**) LSV curves before and after stability measurements of RuO_2_||CMC system.

Based on the outstanding HER and OER performance of ternary CMC catalysts, the OWS activity was also studied via fabricating electrolyzers with configurations of CMC||CMC and RuO_2_||CMC. A digital photograph of the assembled water-splitting electrolyzer is shown in Figure 6a. During electrolysis, vigorous gas bubble evolution was observed at both the anode and cathode, indicating efficient water decomposition. The polarization curves of the OWS devices are shown in Figure 6b. The CMC||CMC-configured electrolyzer achieved a current density of 10 mA cm^−2^ and 1000 mA cm^−2^ at cell voltages of 1.69 V and 2.32 V, respectively. To benchmark the performance, an additional electrolyzer was constructed using RuO_2_/NF as the anode and CMC as the cathode (RuO_2_||CMC) [39,41]. The RuO_2_||CMC electrolyzer demonstrated enhanced performance, requiring only 1.57 V and 2.18 V to reach 10 and1000 mA cm^−2^, respectively (Figure 6b). A comparative summary of the voltages required to reach 10, 100, and 500 mA cm^−2^ for both CMC||CMC and RuO_2_||CMC configurations is presented in Table S4. These results clearly demonstrate the efficacy of the CMC-based systems for large-scale and industrially relevant water-splitting applications. These findings clearly establish the excellent bifunctional catalytic activity and industrial feasibility of the CMC-based systems. The superior performance of the RuO_2_||CMC configurations is attributed to the high conductivity and excellent OER kinetics of RuO_2_, which facilitate rapid charge transfer and reduce the energy barrier for water oxidation, while the CMC electrode provides efficient and stable HER activity. Notably, the RuO_2_||CMC system surpassed the performance of many state-of-the-art catalysts, including Ru_2_Ni_2_SNs/C (1.58 V) [42], Cr–Co_3_S_4_/NiMoS_4_ (1.58 V) [43], CoMo-LDH (1.63 V) [20], O-CoMoS (1.6 V) [44], CoFeZr oxides (1.63 V) [45], Co/CNFs (1.69 V) [46], and N-NiMoO_4_/Ni/CNTs (1.64 V) [47], demonstrating the outstanding efficiency of the developed catalyst pair. To evaluate the long-term durability of the electrolyzer systems, chronoamperometric stability tests were conducted at a constant current density of 100 mA cm^−2^ (without iR compensation), as shown in Figure 6c. Both CMC||CMC and RuO_2_||CMC systems exhibited excellent operational stability over 100 h of continuous operation. In particular, the RuO_2_||CMC electrolyzer maintained a more stable and lower voltage profile throughout the test, without any observable potential drift or performance degradation. The LSV curves recorded before and after the stability test (Figure 6d) remained virtually unchanged for the RuO_2_||CMC electrolyzer, confirming the outstanding structural and electrochemical durability of the electrode materials under prolonged operation. Furthermore, no significant voltage fluctuations were observed even under high current density conditions, confirming the mechanical integrity and robust catalytic performance of the electrolyzer components. These findings strongly indicate that the observed current in both configurations is primarily driven by sustained HER and OER activity, with minimal contributions from side reactions. Overall, the RuO_2_||CMC system demonstrates exceptional bifunctional electrocatalytic activity, excellent long-term durability, and high current-density capability, making it a promising and scalable candidate for practical hydrogen generation through efficient overall water splitting.

## 4. Conclusions

Ternary metal-oxide catalyst electrodes were successfully fabricated on nickel foam substrates using a hydrothermal technique. The composition of each catalyst influenced its surface morphologies, leading to various structural features including microporous-flake composites, ultrathin flakes, microspheres with nanoneedles, and randomly oriented hexagons. Among them, the ternary CMC-based electrode exhibited the highest electrochemical surface area and superior electronic conductivity, resulting in outstanding electrocatalytic activity. Remarkably, the CMC-based electrode achieved excellent HER and OER performance, requiring low overpotentials of 124 mV and 340 mV to reach current densities of −10 mA cm^−2^ and 100 mA cm^−2^, respectively, significantly outperforming the binary counterparts (CM, CC, and MC). Furthermore, the RuO_2_||CMC-configured electrolyzer displayed exceptional overall water splitting performance, achieving a current density of 10 mA cm^−2^ at a low cell voltage of 1.57 V, while maintaining long-term stability in an alkaline electrolyte. The enhanced electrochemical performance is attributed to the increased active surface area and improved charge transfer properties derived from the synergistic interaction among Co, Mo, and Ce. Overall, this work presents a facile route to develop efficient and robust catalyst electrodes for green hydrogen generation via electrolysis.

## Data Availability

The original contributions presented in this study are included in the article/Supplementary Materials. Further inquiries can be directed to the corresponding authors.

## Supplementary Materials

The following supporting information can be downloaded at: https://www.mdpi.com/article/10.3390/ma18174052/s1, Figure S1. Scanning electron microscopic images of the as-prepared (a) CMC, (b) CM, (c) CC, and (d) MC; Figure S2. (a) EDS analysis spectra of the ternary CMC catalyst electrode, suggesting the presence of the Co, Mo, Ce, and O in the sample. (b) overall EDS elemental mapping image of the CMC catalyst; Figure S3. XPS survey spectra of the ternary CMC catalyst; Table S1. HER overpotential of all the samples obtained at different current densities of the −10, −500, and −1000 mA cm^−2^ and their Tafel slopes; Figure S4. HER polarization curves of the ternary CMC catalyst electrode before and after stability measurements of 100 h at a current density of 100 mA cm^−2^; Table S2. OER overpotential of all the samples obtained at different current densities of the 10, 500, and 100 mA cm^−2^ and their Tafel slopes; Figure S5. OER polarization curves of the ternary CMC catalyst before and after stability measurements of 100 h at a current density of 100 mA cm^−2^; Figure S6. CV curves of the catalyst electrodes recorded in non-faradic region at different scan rates of 50-140 mVs^−1^: (a) CM (b) CC, and (c) MC; Table S3. Electrochemical Cdl and ECSA parameters obtained from CV analysis; Table S4. OWS activity of the CMC-based electrolyzers and their required cell voltages at different current densities.
