# Supplementary material for: Design of CoMoCe-Oxide Nanostructured Composites as Robust Bifunctional Electrocatalyst for Water Electrolysis Overall Efficiency
Source: Materials (Basel). 2025 Aug 29;18(17):4052. doi: 10.3390/ma18174052 (PMC12428978; doi:10.3390/ma18174052)
Supplement: Supplementary file 1 [file materials-18-04052-s001.zip › materials-3808016-supplementary.pdf]

Supplementary Information

Design of CoMoCe Oxide Nanostructured Composites as Robust Bifunctional Electrocatalyst for Efficient Overall Water Electrolysis

Akbar I. Inamdar*[1], Amol S. Salunke[2], Jyoti V. Patil[3,4], Sawanta S. Mali[3], Chang Kook Hong[3,4], Basit Ali[5], Supriya A. Patil[6], Nabeen K. Shrestha[1], Sejoon Lee *[1], Sangeun Cho*[1]

[1]Division of System Semiconductor, Dongguk University, Seoul, 04620, Republic of Korea

[2]Department of Semiconductor Science, Dongguk University, Seoul 04620, Republic of Korea

[3]Polymer Energy Materials Laboratory, School of Chemical Engineering, Chonnam National University, Gwangju, 61186, South Korea

[4]Optoelectronic Convergence Research Center School of Chemical Engineering Chonnam National University Gwangju 61186, South Korea

[5]Department of Chemistry and Materials Science, School of Chemical Engineering, Aalto University, P.O. Box 16100, Aalto, FI-00076, Finland

[6]Department of Nanotechnology and Advanced Materials Engineering, Sejong University, Seoul 05006, Republic of Korea

***Corresponding Authors**

E-mail addresses: akbarphysics2002@gmail.com (A. I. I.), sejoon@dongguk.edu (S. L.)

sangeun.c@dongguk.edu (S. C.)

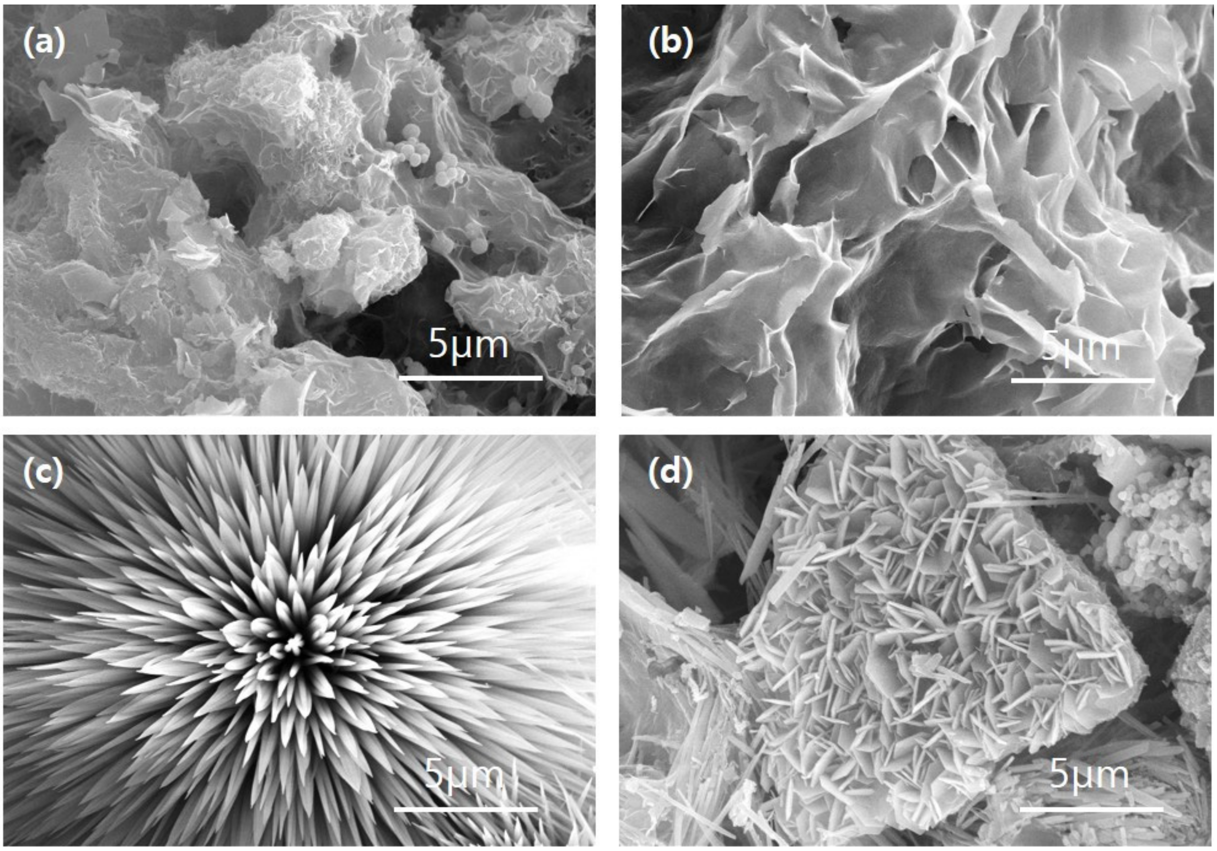

Figure S1. Scanning electron microscopic images of the as-prepared **(a)** CMC, **(b)** CM, **(c)** CC, and **(d)** MC.

(a)

(b)

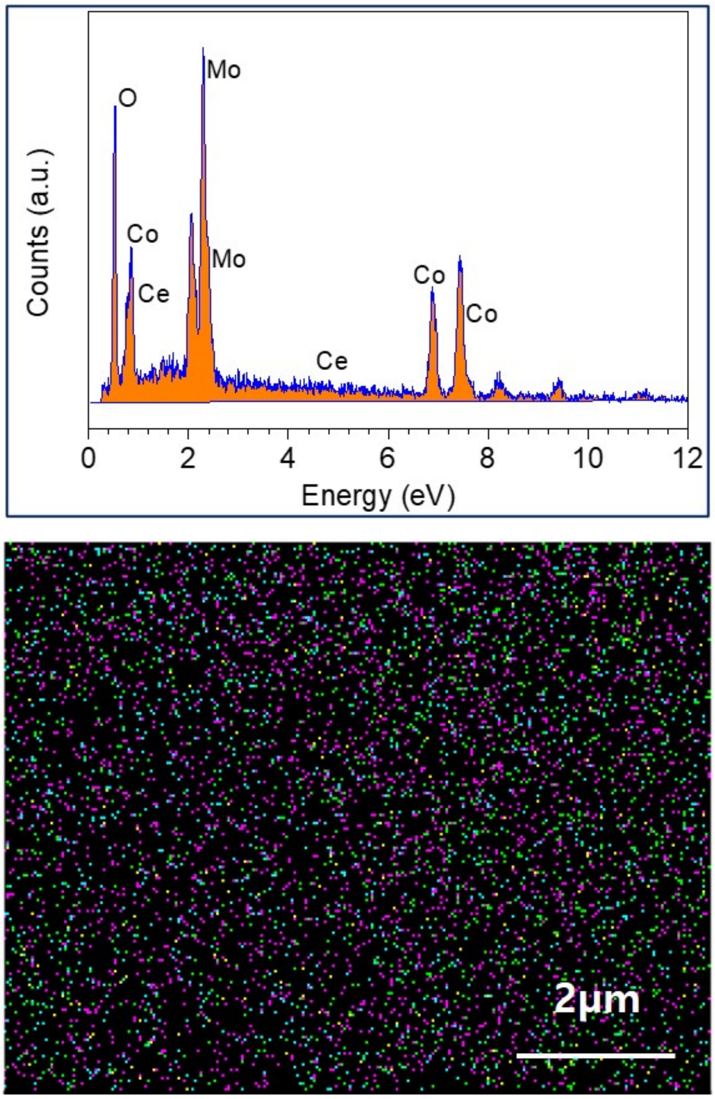

Figure S2. (a) EDS analysis spectra of the ternary CMC catalyst electrode, suggesting the presence of the Co, Mo, Ce, and O in the sample. **(b)** overall EDS elemental mapping image of the CMC catalyst.

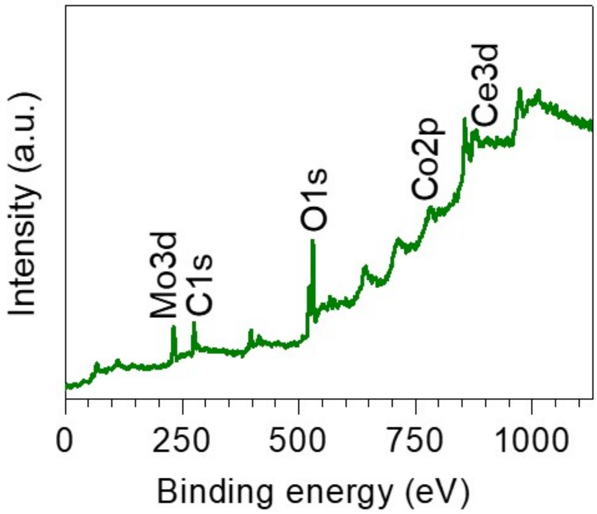

Figure S3. XPS survey spectra of the ternary CMC catalyst.

Table S1. HER overpotential of all the samples obtained at different current densities of the −10, −500, and −1000 mA cm^{-2} and their Tafel slopes.

Sample	HER @ −10 mA cm^{-2}	@−500 mA cm^{-2}	@−1000 mA cm^{-2}	Tafel slope mV dec^{-1}
**CMC**	**124**	**347**	**407**	**115.5**
**CM**	149	378	458	125.7
**CC**	178	457	566	131.9
**MC**	162	433	545	192.0

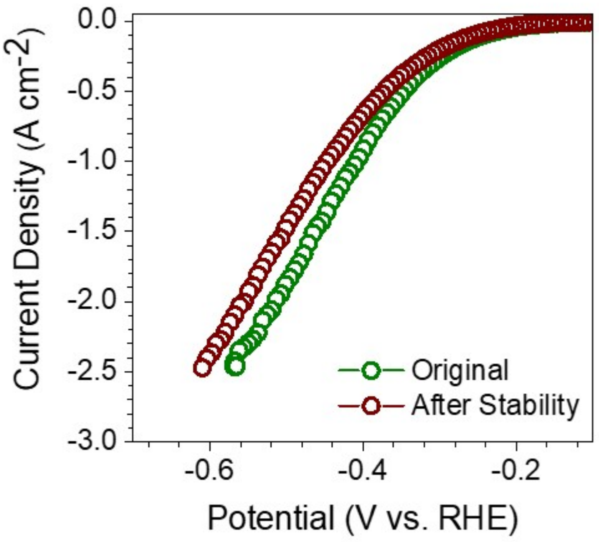

Figure S4. HER polarization curves of the ternary CMC catalyst electrode before and after stability measurements of 100 h at a current density of 100 mA cm^{-2}.

Table S2. OER overpotential of all the samples obtained at different current densities of the 10, 500, and 100 mA cm^{-2} and their Tafel slopes.

Sample	OER @ 10 mA cm^{-2}	@500 mA cm^{-2}	@1000 mA cm^{-2}	Tafel slope mV dec^{-1}
**CMC**	**340**	**436**	**496**	**118.08**
**CM**	411	488	529	136.6
**CC**	365	485	575	134.02
**MC**	444	578	-	141.3

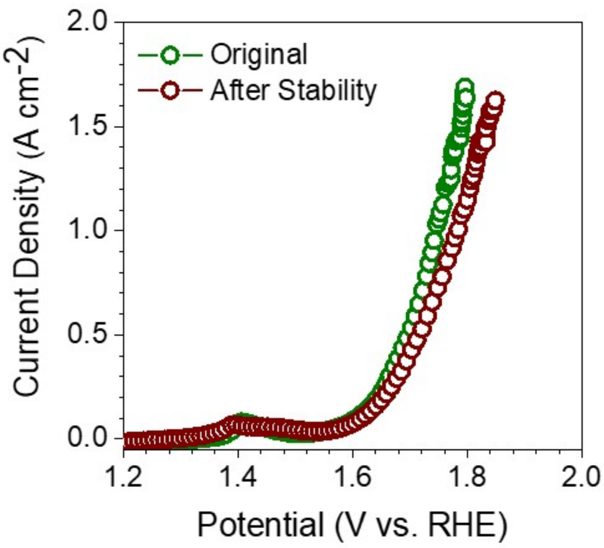

Figure S5. OER polarization curves of the ternary CMC catalyst before and after stability measurements of 100 h at a current density of 100 mA cm^{-2}.

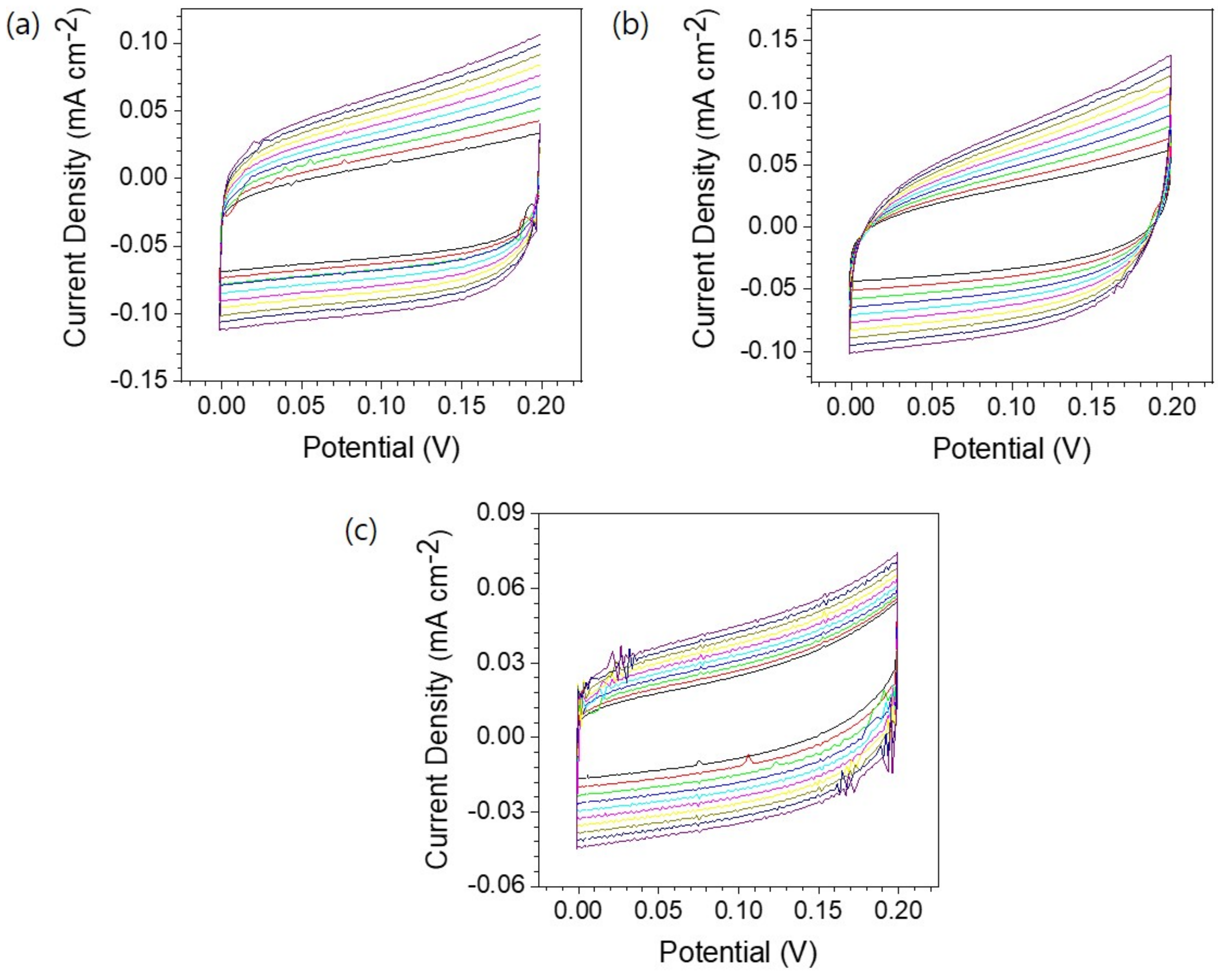

Figure S6. CV curves of the catalyst electrodes recorded in non-faradic region at different scan rates of 50-140 mVs^{-1}: **(a)** CM **(b)** CC, and **(c)** MC.

Table S3. Electrochemical C_{dl} and ECSA parameters obtained from CV analysis.

Sample	C_{dl} μF cm^{-2}	ECSA
**CMC**	**1.21**	**30.25**
**CM**	0.67	16.75
**CC**	0.65	16.25
**MC**	0.22	5.5

Table S4. OWS activity of the CMC-based electrolyzers and their required cell voltages at different current densities.

Sample	Cell voltage @ 10 mA cm^{-2}	@100 mA cm^{-2}	@500 mA cm^{-2}	@1000 mA cm^{-2}
**CMC‖CMC**	1.69	1.93	2.16	2.32
**RuO_2‖CMC**	1.57	1.83	2.04	2.18

Turnover frequency

TOF = j x A / N x n x F

Geometric area = **1.0 cm²** (currents are per cm²)

Surface site density = Γ = 1 site\nm^{-2} =1.66054 × 10^{-10} mol\cm^{-2}

Faraday constant F = 96485 C\mol^{-1}

Currents: HER I = 10 mA = 0.010 A ; OER I = 100 mA = 0.100 A

Product formation rates (same for every sample)

$$rH2 = I(HER)/2F = 5.182\times10^{-8}\ mol\backslash s^{-1}$$

$$rO2 = I(OER)/4F = 2.591\times10^{-7}\ mol\backslash s^{-1}$$

Active-site moles: Nsites = $\Gamma\times$ ECSA

$$N = ECSA \times 1.66054\times10^{-10}\ mol/cm^2$$

CoMoCe (HER) = TOF = 0.015 * 1 / 5.021 * 10^{-9} * 2 * 96485 = 10.32 S^{-1}

**Sample (HER)** (**-0.15V vs RHE**)	**mA**	**Amp**
CoMoCe	-15.46	0.01546
CoMo	-10.12	0.01012
CoCe	-8.099	0.008
MoCe	-5.522	0.0055

**Sample(OER) (1.65 V vs RHE)**	**mA**	**Amp**
CoMoCe	393.66	0.393
CoMo	120.62	0.120
CoCe	241.97	0.2419
MoCe	67.53	0.0675

TOF =

**Sample**	**ECSA**	**Nsite ($*10^{-9}$)**	**TOF(HER)**	**TOF(OER)**
CoMoCe	30.25	5.02	15.48	201.34
CoMo	16.75	2.78	10.44	61.95
CoCe	16.25	2.70	5.67	124.4
MoCe	5.50	0.913	8.25	34.58
